# 3-Hy­droxy-2-(4-meth­oxy­benzene­sulfonamido)­butanoic acid

**DOI:** 10.1107/S1600536811046502

**Published:** 2011-11-12

**Authors:** Suman Sinha, Hasnah Osman, Habibah A Wahab, Madhukar Hemamalini, Hoong-Kun Fun

**Affiliations:** a School of Pharmaceutical Sciences, Universiti Sains Malaysia, 11800 USM, Penang, Malaysia; b School of Chemical Sciences, Universiti Sains Malaysia, 11800 USM, Penang, Malaysia; c X-ray Crystallography Unit, School of Physics, Universiti Sains Malaysia, 11800 USM, Penang, Malaysia

## Abstract

The title compound, C_11_H_15_NO_6_S, features a distorted tetra­hedral geometry for the S atom. One of the sulfonamide O atoms is approximately coplanar with the benzene ring [C—C—S—O torsion angle = −160.81 (7)°], whereas the other lies well below the plane [C—C—S—O = −29.66 (8)°]. In the crystal, O—H⋯O and C—H⋯O hydrogen bonds link the mol­ecules into chains parallel to the *b* axis.

## Related literature

For details and applications of sulfonamides, see: Supuran *et al.* (2003 ▶); Scozzafava *et al.* (2003 ▶); Robinson *et al.* (2003 ▶); Delaet *et al.* (2003 ▶). For the stability of the temperature controller used in the data collection, see: Cosier & Glazer (1986 ▶).
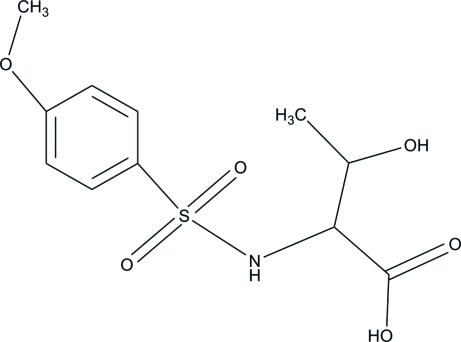


## Experimental

### 

#### Crystal data

C_11_H_15_NO_6_S
*M* *_r_* = 289.30
Orthorhombic, 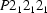

*a* = 5.6505 (2) Å
*b* = 9.9204 (3) Å
*c* = 23.0561 (6) Å
*V* = 1292.41 (7) Å^3^
*Z* = 4
Mo *K*α radiation
μ = 0.27 mm^−1^
*T* = 100 K
0.75 × 0.19 × 0.17 mm

#### Data collection

Bruker SMART APEXII CCD area-detector diffractometer
Absorption correction: multi-scan (*SADABS*; Bruker, 2009 ▶) *T*_min_ = 0.821, *T*_max_ = 0.954
35154 measured reflections
5756 independent reflections
5505 reflections with *I* > 2σ(*I*)
*R*_int_ = 0.027

#### Refinement

*R*[*F* ^2^ > 2σ(*F* ^2^)] = 0.028
*wR*(*F* ^2^) = 0.073
*S* = 1.07
5756 reflections
186 parameters
H atoms treated by a mixture of independent and constrained refinement
Δρ_max_ = 0.34 e Å^−3^
Δρ_min_ = −0.42 e Å^−3^
Absolute structure: Flack (1983 ▶), 2444 Friedel pairs
Flack parameter: 0.02 (4)

### 

Data collection: *APEX2* (Bruker, 2009 ▶); cell refinement: *SAINT* (Bruker, 2009 ▶); data reduction: *SAINT*; program(s) used to solve structure: *SHELXTL* (Sheldrick, 2008 ▶); program(s) used to refine structure: *SHELXTL*; molecular graphics: *SHELXTL*; software used to prepare material for publication: *SHELXTL* and *PLATON* (Spek, 2009 ▶).

## Supplementary Material

Crystal structure: contains datablock(s) global, I. DOI: 10.1107/S1600536811046502/rz2664sup1.cif

Structure factors: contains datablock(s) I. DOI: 10.1107/S1600536811046502/rz2664Isup2.hkl

Supplementary material file. DOI: 10.1107/S1600536811046502/rz2664Isup3.cml

Additional supplementary materials:  crystallographic information; 3D view; checkCIF report

## Figures and Tables

**Table 1:** Hydrogen-bond geometry (Å, °)

*D*—H⋯*A*	*D*—H	H⋯*A*	*D*⋯*A*	*D*—H⋯*A*
O5—H1*O*5⋯O6^i^	0.844 (18)	1.816 (19)	2.6019 (10)	154.3 (17)
O6—H1*O*6⋯O3^i^	0.81 (2)	1.99 (2)	2.7990 (10)	174.2 (19)
C5—H5*A*⋯O4^ii^	0.93	2.52	3.3732 (11)	153
C8—H8*A*⋯O2^iii^	0.98	2.48	3.4000 (11)	156

Symmetry codes: (i) 
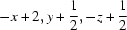
; (ii) 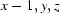; (iii) 
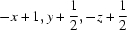
.

**Fig. 1.**
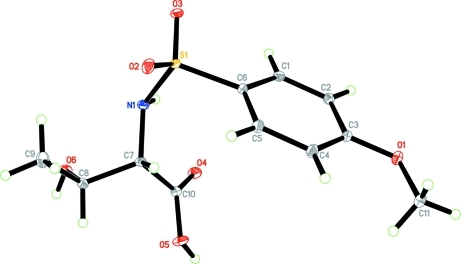
The asymmetric unit of the title compound, showing 30% probability displacement ellipsoids.

**Fig. 2.**
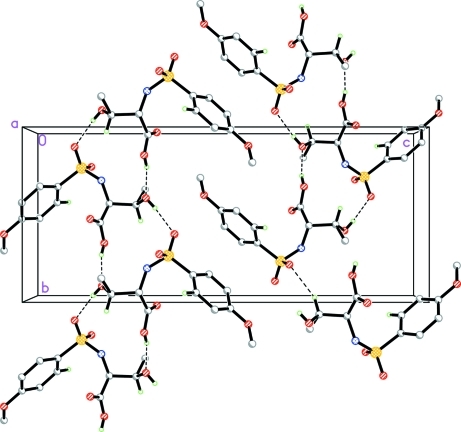
The crystal packing of the title compound viewed along the a axis. H atoms not involved in hydrogen bonding (dashed lines) are omitted.

## supplementary crystallographic information

### Comment

The chemistry of sulfonamides is of interest as they show distinct physical, chemical and biological properties. Sulfonamides are used as anticancer, anti-inflammatory and antiviral agents (Supuran *et al.*, 2003; Scozzafava *et al.*, 2003). Amino acid-derived sulfonamides are shown to be active against Procollagen C-terminal protease, which is a member of the metzincin enzyme family (Robinson *et al.*, 2003; Delaet *et al.*, 2003).

The asymmetric unit of the title compound is shown in Fig. 1. The S atom is tetrahedrally bonded within a CNO_2_ donor set with the greatest deviation manifested in the O2—S1—O3 angle of 120.08 (5)°. The sulfonamide O2 atom is approximately co-planar with the benzene ring [the O2-S1-C1-C6 torsion angle is -160.81 (7)°] whereas the O3 atom lies well below the plane [O3-S1-C1-C6 = -29.66 (8)°].

In the crystal structure (Fig. 2), intermolecular O—H···O and C—H···O hydrogen bonds (Table 1) link the molecules into chains parallel to the *b* axis.

### Experimental

To a solution of L-threonine (3 mmol, 0.618 g) in distilled water (10 ml), 4-methoxybenzene sulphonyl chloride (3 mmol, 0.357 g) was suspended. The pH of the solution was maintained at 8 by continuously adding 1M sodium carbonate solution throughout the reaction at room temperature. After the completion of the reaction, the pH was adjusted to 2 using 1N HCl solution which resulted in the formation of the precipitate which was filtered, dried and recrystallized in methanol to yield the title compound.

### Refinement

Atoms H1N1, H1O5 and H1O6 were located from a difference Fourier map and refined freely [N–H = 0.887 (17) and O–H = 0.81 (2)–0.845 (19) Å]. The remaining H atoms were positioned geometrically [C–H = 0.93–0.98 Å] and were refined using a riding model, with *U*_iso_(H) = 1.2 or 1.5 *U*_eq_(C). A rotating group model was applied to the methyl groups. 2444 Friedel pairs were used to determine the absolute configuration.

### Crystal data

**Table:**

C_11_H_15_NO_6_S	*F*(000) = 608
*M**_r_* = 289.30	*D*_x_ = 1.487 Mg m^−^^3^
Orthorhombic, *P*2_1_2_1_2_1_	Mo *K*α radiation, λ = 0.71073 Å
Hall symbol: P 2ac 2ab	Cell parameters from 9845 reflections
*a* = 5.6505 (2) Å	θ = 3.4–35.2°
*b* = 9.9204 (3) Å	µ = 0.27 mm^−^^1^
*c* = 23.0561 (6) Å	*T* = 100 K
*V* = 1292.41 (7) Å^3^	Block, colourless
*Z* = 4	0.75 × 0.19 × 0.17 mm

### Data collection

**Table:**

Bruker SMART APEXII CCD area-detector diffractometer	5756 independent reflections
Radiation source: fine-focus sealed tube	5505 reflections with *I* > 2σ(*I*)
graphite	*R*_int_ = 0.027
φ and ω scans	θ_max_ = 35.4°, θ_min_ = 2.2°
Absorption correction: multi-scan (*SADABS*; Bruker, 2009)	*h* = −7→9
*T*_min_ = 0.821, *T*_max_ = 0.954	*k* = −16→16
35154 measured reflections	*l* = −37→37

### Refinement

**Table:**

Refinement on *F*^2^	Secondary atom site location: difference Fourier map
Least-squares matrix: full	Hydrogen site location: inferred from neighbouring sites
*R*[*F*^2^ > 2σ(*F*^2^)] = 0.028	H atoms treated by a mixture of independent and constrained refinement
*wR*(*F*^2^) = 0.073	*w* = 1/[σ^2^(*F*_o_^2^) + (0.0407*P*)^2^ + 0.163*P*] where *P* = (*F*_o_^2^ + 2*F*_c_^2^)/3
*S* = 1.07	(Δ/σ)_max_ = 0.001
5756 reflections	Δρ_max_ = 0.34 e Å^−^^3^
186 parameters	Δρ_min_ = −0.42 e Å^−^^3^
0 restraints	Absolute structure: Flack (1983), 2444 Friedel pairs
Primary atom site location: structure-invariant direct methods	Flack parameter: 0.02 (4)

### Special details

**Table:**

Experimental. The crystal was placed in the cold stream of an Oxford Cryosystems Cobra open-flow nitrogen cryostat (Cosier & Glazer, 1986) operating at 100.0 (1) K.
Geometry. All s.u.'s (except the s.u. in the dihedral angle between two l.s. planes) are estimated using the full covariance matrix. The cell s.u.'s are taken into account individually in the estimation of s.u.'s in distances, angles and torsion angles; correlations between s.u.'s in cell parameters are only used when they are defined by crystal symmetry. An approximate (isotropic) treatment of cell s.u.'s is used for estimating s.u.'s involving l.s. planes.
Refinement. Refinement of F^2^ against ALL reflections. The weighted R-factor wR and goodness of fit S are based on F^2^, conventional R-factors R are based on F, with F set to zero for negative F^2^. The threshold expression of F^2^ > 2σ(F^2^) is used only for calculating R-factors(gt) etc. and is not relevant to the choice of reflections for refinement. R-factors based on F^2^ are statistically about twice as large as those based on F, and R- factors based on ALL data will be even larger.

### Fractional atomic coordinates and isotropic or equivalent isotropic displacement parameters (Å^2^)

**Table:**

	*x*	*y*	*z*	*U*_iso_*/*U*_eq_	
S1	0.72919 (4)	0.22850 (2)	0.138337 (9)	0.01332 (4)	
O1	0.78573 (15)	0.61425 (7)	−0.05450 (3)	0.02057 (13)	
O2	0.48689 (13)	0.21583 (8)	0.15597 (3)	0.01902 (13)	
O3	0.86556 (14)	0.10991 (7)	0.12458 (3)	0.01897 (13)	
O4	1.15328 (13)	0.52453 (7)	0.17150 (3)	0.02082 (14)	
O5	0.85821 (13)	0.66432 (7)	0.19810 (4)	0.01992 (14)	
O6	0.96165 (12)	0.40568 (7)	0.30315 (3)	0.01456 (11)	
N1	0.87145 (14)	0.30287 (7)	0.19112 (3)	0.01372 (12)	
C1	0.93213 (15)	0.33833 (9)	0.04102 (4)	0.01481 (14)	
H1A	1.0550	0.2771	0.0462	0.018*	
C2	0.94041 (16)	0.43159 (9)	−0.00357 (4)	0.01560 (14)	
H2A	1.0688	0.4326	−0.0288	0.019*	
C3	0.75624 (17)	0.52429 (8)	−0.01084 (3)	0.01491 (14)	
C4	0.56040 (16)	0.52178 (10)	0.02592 (4)	0.01660 (15)	
H4A	0.4368	0.5824	0.0206	0.020*	
C5	0.55119 (15)	0.42778 (9)	0.07069 (4)	0.01495 (14)	
H5A	0.4214	0.4255	0.0955	0.018*	
C6	0.73634 (15)	0.33731 (8)	0.07829 (3)	0.01262 (12)	
C7	0.78523 (15)	0.43593 (8)	0.20919 (4)	0.01261 (13)	
H7A	0.6333	0.4522	0.1899	0.015*	
C8	0.74478 (15)	0.44309 (8)	0.27510 (3)	0.01324 (13)	
H8A	0.7047	0.5359	0.2858	0.016*	
C9	0.54836 (17)	0.35081 (11)	0.29434 (4)	0.02040 (17)	
H9A	0.5260	0.3595	0.3354	0.031*	
H9B	0.5891	0.2593	0.2852	0.031*	
H9C	0.4047	0.3748	0.2747	0.031*	
C10	0.95658 (16)	0.54537 (9)	0.19018 (4)	0.01371 (14)	
C11	0.6019 (2)	0.71196 (10)	−0.06293 (4)	0.02317 (19)	
H11A	0.6467	0.7728	−0.0934	0.035*	
H11B	0.5782	0.7616	−0.0277	0.035*	
H11C	0.4578	0.6668	−0.0734	0.035*	
H1N1	1.025 (3)	0.3010 (15)	0.1834 (7)	0.025 (4)*	
H1O5	0.952 (3)	0.7296 (19)	0.1933 (8)	0.039 (5)*	
H1O6	1.008 (4)	0.462 (2)	0.3261 (9)	0.059 (6)*	

### Atomic displacement parameters (Å^2^)

**Table:**

	*U*^11^	*U*^22^	*U*^33^	*U*^12^	*U*^13^	*U*^23^
S1	0.01979 (8)	0.00881 (7)	0.01137 (8)	−0.00184 (7)	−0.00180 (7)	0.00073 (6)
O1	0.0300 (3)	0.0169 (3)	0.0149 (3)	0.0028 (3)	0.0028 (3)	0.0051 (2)
O2	0.0214 (3)	0.0182 (3)	0.0175 (3)	−0.0080 (3)	0.0000 (2)	0.0032 (2)
O3	0.0322 (4)	0.0089 (2)	0.0157 (3)	0.0027 (3)	−0.0047 (3)	−0.0007 (2)
O4	0.0202 (3)	0.0154 (3)	0.0269 (3)	0.0005 (2)	0.0100 (3)	0.0002 (3)
O5	0.0183 (3)	0.0094 (3)	0.0321 (4)	0.0004 (2)	0.0039 (3)	0.0003 (3)
O6	0.0156 (2)	0.0127 (3)	0.0154 (3)	−0.0003 (2)	−0.0030 (2)	−0.0036 (2)
N1	0.0178 (3)	0.0104 (3)	0.0130 (3)	0.0009 (2)	−0.0027 (2)	−0.0016 (2)
C1	0.0166 (3)	0.0127 (3)	0.0151 (3)	0.0023 (3)	0.0001 (3)	−0.0012 (3)
C2	0.0184 (3)	0.0147 (3)	0.0137 (3)	0.0010 (3)	0.0032 (3)	−0.0002 (3)
C3	0.0209 (3)	0.0123 (3)	0.0115 (3)	0.0005 (3)	0.0006 (3)	0.0010 (2)
C4	0.0181 (4)	0.0163 (4)	0.0153 (4)	0.0037 (3)	0.0000 (3)	0.0035 (3)
C5	0.0151 (3)	0.0158 (3)	0.0139 (3)	0.0013 (3)	0.0007 (3)	0.0014 (3)
C6	0.0159 (3)	0.0107 (3)	0.0113 (3)	0.0000 (3)	−0.0006 (3)	0.0007 (2)
C7	0.0147 (3)	0.0097 (3)	0.0135 (3)	0.0002 (3)	−0.0003 (3)	−0.0006 (2)
C8	0.0129 (3)	0.0135 (3)	0.0133 (3)	0.0009 (3)	0.0010 (3)	−0.0013 (2)
C9	0.0155 (3)	0.0252 (4)	0.0206 (4)	−0.0034 (3)	0.0036 (3)	0.0027 (3)
C10	0.0163 (3)	0.0113 (3)	0.0136 (3)	0.0005 (3)	0.0007 (3)	0.0002 (3)
C11	0.0355 (5)	0.0165 (4)	0.0175 (4)	0.0033 (4)	−0.0036 (4)	0.0037 (3)

### Geometric parameters (Å, °)

**Table:**

S1—O2	1.4337 (8)	C2—H2A	0.9300
S1—O3	1.4416 (7)	C3—C4	1.3940 (13)
S1—N1	1.6345 (8)	C4—C5	1.3922 (12)
S1—C6	1.7561 (8)	C4—H4A	0.9300
O1—C3	1.3555 (10)	C5—C6	1.3895 (12)
O1—C11	1.4338 (13)	C5—H5A	0.9300
O4—C10	1.2097 (11)	C7—C10	1.5193 (12)
O5—C10	1.3171 (11)	C7—C8	1.5385 (11)
O5—H1O5	0.845 (19)	C7—H7A	0.9800
O6—C8	1.4344 (11)	C8—C9	1.5055 (13)
O6—H1O6	0.81 (2)	C8—H8A	0.9800
N1—C7	1.4674 (11)	C9—H9A	0.9600
N1—H1N1	0.887 (17)	C9—H9B	0.9600
C1—C2	1.3839 (13)	C9—H9C	0.9600
C1—C6	1.4008 (12)	C11—H11A	0.9600
C1—H1A	0.9300	C11—H11B	0.9600
C2—C3	1.3989 (13)	C11—H11C	0.9600
			
O2—S1—O3	120.08 (5)	C1—C6—S1	120.41 (6)
O2—S1—N1	107.35 (4)	N1—C7—C10	110.45 (7)
O3—S1—N1	105.62 (4)	N1—C7—C8	111.80 (7)
O2—S1—C6	107.42 (4)	C10—C7—C8	110.28 (7)
O3—S1—C6	108.41 (4)	N1—C7—H7A	108.1
N1—S1—C6	107.35 (4)	C10—C7—H7A	108.1
C3—O1—C11	117.17 (8)	C8—C7—H7A	108.1
C10—O5—H1O5	113.7 (13)	O6—C8—C9	109.86 (7)
C8—O6—H1O6	113.2 (16)	O6—C8—C7	107.85 (7)
C7—N1—S1	117.01 (6)	C9—C8—C7	111.87 (7)
C7—N1—H1N1	113.6 (10)	O6—C8—H8A	109.1
S1—N1—H1N1	108.9 (10)	C9—C8—H8A	109.1
C2—C1—C6	119.16 (8)	C7—C8—H8A	109.1
C2—C1—H1A	120.4	C8—C9—H9A	109.5
C6—C1—H1A	120.4	C8—C9—H9B	109.5
C1—C2—C3	120.23 (8)	H9A—C9—H9B	109.5
C1—C2—H2A	119.9	C8—C9—H9C	109.5
C3—C2—H2A	119.9	H9A—C9—H9C	109.5
O1—C3—C4	124.11 (8)	H9B—C9—H9C	109.5
O1—C3—C2	115.49 (8)	O4—C10—O5	126.17 (9)
C4—C3—C2	120.39 (8)	O4—C10—C7	124.47 (8)
C5—C4—C3	119.50 (8)	O5—C10—C7	109.35 (7)
C5—C4—H4A	120.2	O1—C11—H11A	109.5
C3—C4—H4A	120.2	O1—C11—H11B	109.5
C6—C5—C4	119.87 (8)	H11A—C11—H11B	109.5
C6—C5—H5A	120.1	O1—C11—H11C	109.5
C4—C5—H5A	120.1	H11A—C11—H11C	109.5
C5—C6—C1	120.84 (8)	H11B—C11—H11C	109.5
C5—C6—S1	118.63 (6)		
			
O2—S1—N1—C7	−57.73 (7)	O3—S1—C6—C5	154.30 (7)
O3—S1—N1—C7	173.03 (6)	N1—S1—C6—C5	−92.03 (7)
C6—S1—N1—C7	57.50 (7)	O2—S1—C6—C1	−160.81 (7)
C6—C1—C2—C3	0.59 (13)	O3—S1—C6—C1	−29.66 (8)
C11—O1—C3—C4	−0.22 (13)	N1—S1—C6—C1	84.01 (8)
C11—O1—C3—C2	−179.31 (8)	S1—N1—C7—C10	−108.33 (7)
C1—C2—C3—O1	177.73 (8)	S1—N1—C7—C8	128.47 (6)
C1—C2—C3—C4	−1.40 (14)	N1—C7—C8—O6	55.38 (9)
O1—C3—C4—C5	−177.88 (9)	C10—C7—C8—O6	−67.91 (8)
C2—C3—C4—C5	1.17 (14)	N1—C7—C8—C9	−65.53 (9)
C3—C4—C5—C6	−0.15 (14)	C10—C7—C8—C9	171.18 (7)
C4—C5—C6—C1	−0.66 (13)	N1—C7—C10—O4	−11.26 (12)
C4—C5—C6—S1	175.37 (7)	C8—C7—C10—O4	112.81 (10)
C2—C1—C6—C5	0.43 (13)	N1—C7—C10—O5	169.63 (7)
C2—C1—C6—S1	−175.52 (7)	C8—C7—C10—O5	−66.30 (9)
O2—S1—C6—C5	23.15 (8)		

### Hydrogen-bond geometry (Å, °)

**Table:**

*D*—H···*A*	*D*—H	H···*A*	*D*···*A*	*D*—H···*A*
O5—H1O5···O6^i^	0.844 (18)	1.816 (19)	2.6019 (10)	154.3 (17)
O6—H1O6···O3^i^	0.81 (2)	1.99 (2)	2.7990 (10)	174.2 (19)
C5—H5A···O4^ii^	0.93	2.52	3.3732 (11)	153.
C8—H8A···O2^iii^	0.98	2.48	3.4000 (11)	156.

Symmetry codes: (i) −*x*+2, *y*+1/2, −*z*+1/2; (ii) *x*−1, *y*, *z*; (iii) −*x*+1, *y*+1/2, −*z*+1/2.
